# Potential biomarkers uncovered by bioinformatics analysis in sotorasib resistant-pancreatic ductal adenocarcinoma

**DOI:** 10.3389/fmed.2023.1107128

**Published:** 2023-06-15

**Authors:** Prasanna Srinivasan Ramalingam, Annadurai Priyadharshini, Isaac Arnold Emerson, Sivakumar Arumugam

**Affiliations:** ^1^Protein Engineering Lab, School of Biosciences and Technology, Vellore Institute of Technology, Vellore, India; ^2^Bioinformatics Programming Laboratory, Department of Biotechnology, School of Biosciences and Technology, Vellore Institute of Technology, Vellore, Tamil Nadu, India

**Keywords:** sotorasib, KRAS G12C inhibitor, resistance, pancreatic ductal adenocarcinoma, ribosomal proteins, precision medicine

## Abstract

**Background:**

Mutant KRAS-induced tumorigenesis is prevalent in lung, colon, and pancreatic ductal adenocarcinomas. For the past 3 decades, KRAS mutants seem undruggable due to their high-affinity GTP-binding pocket and smooth surface. Structure-based drug design helped in the design and development of first-in-class KRAS G12C inhibitor sotorasib (AMG 510) which was then approved by the FDA. Recent reports state that AMG 510 is becoming resistant in non-small-cell lung cancer (NSCLC), pancreatic ductal adenocarcinoma (PDAC), and lung adenocarcinoma patients, and the crucial drivers involved in this resistance mechanism are unknown.

**Methods:**

In recent years, RNA-sequencing (RNA-seq) data analysis has become a functional tool for profiling gene expression. The present study was designed to find the crucial biomarkers involved in the sotorasib (AMG 510) resistance in KRAS G12C-mutant MIA-PaCa2 cell pancreatic ductal adenocarcinoma cells. Initially, the GSE dataset was retrieved from NCBI GEO, pre-processed, and then subjected to differentially expressed gene (DEG) analysis using the limma package. Then the identified DEGs were subjected to protein–protein interaction (PPI) using the STRING database, followed by cluster analysis and hub gene analysis, which resulted in the identification of probable markers.

**Results:**

Furthermore, the enrichment and survival analysis revealed that the small unit ribosomal protein (RP) RPS3 is the crucial biomarker of the AMG 510 resistance in KRAS G12C-mutant MIA-PaCa2 cell pancreatic ductal adenocarcinoma cells.

**Conclusion:**

Finally, we conclude that RPS3 is a crucial biomarker in sotorasib resistance which evades apoptosis by MDM2/4 interaction. We also suggest that the combinatorial treatment of sotorasib and RNA polymerase I machinery inhibitors could be a possible strategy to overcome resistance and should be studied in *in vitro* and *in vivo* settings in near future.

## Graphical Abstract

Study outcomes and proposed future directions to combat sotorasib resistance in KRAS G12C mutant cells. 
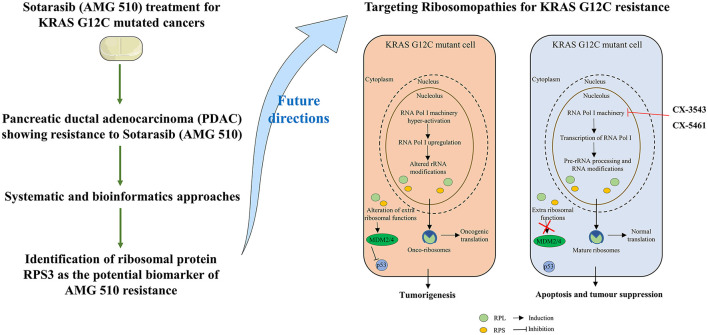

## 1. Introduction

Mutant RAS-harboring cancers are predominant in many cancers including pancreatic, breast, colon, and lung, which corresponds to nearly 30% of all cancers (1, 2). Unlike NRAS and HRAS isoforms of RAS, the KRAS isoform has high mutation frequencies at mutational hotspots G12 (89%), G13 (9%), and Q61 (1%) residues (3–5). Overall, the G12th residue is the most mutated position of KRAS with G12D as the most prevalent mutation with 36%, followed by the G12V and G12C mutations with 23 and 14%, respectively (6). KRAS is a small GTPase that acts as a molecular switch by GTP-bound (active form) and GDP-bound (inactive form) states and triggers the downstream signal transduction pathways (7, 8). The GDP to GTP conversion is mediated by the guanine nucleotide exchange factors (GEFs), and the GTP to GDP hydrolysis is mediated by GTPase-activating proteins (GAPs) (9, 10). The mutant KRAS maintains the GTP-bound active state and overcomes the GTPase activity and initiates nearly 80 different downstream effector signaling pathways including MAPK and PI3K-mTOR signaling which further activates JUN and MYC transcription factors and promotes the cancer cell survival and proliferation (11–15).

Several strategies have been carried out to inhibit the mutant KRAS signaling such as targeting the upstream effectors (EGFR inhibitors, FGFR1 inhibitors, and IGF1R inhibitors); targeting the inhibitors of KRAS regulators (SOS1 inhibitors and SHP2 inhibitors); direct targeting of KRAS (KRAS on state and off-state inhibitors); downstream effector inhibitors (PI3K inhibitors, mTOR inhibitors, and MEK inhibitors); and cell cycle arrest (CDK4/6 inhibitors) (16–19). Moreover, targeting the other mediators and effectors in the MAPK pathway result in the signaling crosstalk such as MEK-PI3K, RAF-AKT, RAS-SKF, RAS-YAP, and SHP2-dependent MAPK reactivation and SHP2-independent PI3K reactivation (20–22). All the strategies have shown significant outcomes, but the complete inhibition of KRAS was promising in the direct targeting strategy. In general, the intracellular levels of GTP are in micromolar (μM) ranges, and its binds with picomolar (pM) affinity to the GTP-binding pocket of the KRAS, which challenges it as undruggable to the medicinal chemistry and drug discovery researchers to design and develop a potent KRAS mutant small molecule inhibitors (23–25). Finally, the undruggable became druggable by the successful discovery and FDA approval of KRAS G12C inhibitor sotorasib (AMG 510) for the treatment of non-small-cell lung cancer (NSCLC) and other solid tumors (26–28). The sotorasib specifically targets the cryptic pocket of the KRAS G12C (H95/Y96/Q99) and forms the covalent bond with the reactive cysteine at the 12th position, which also limits its ability to target other KRAS mutants such as G12D and G12V that lacks reactive cysteine (29). Recently, in December 2022, FDA granted the accelerated approval for adagrasib (MRTX849) for the treatment of KRAS G12C-mutated NSCLC (30).

Accumulating pieces of evidence report that sotorasib is becoming resistant among NSCLC, pancreatic ductal adenocarcinoma, and colorectal adenocarcinoma patients bearing KRAS G12C mutation and even resulting in hepatotoxicity (31, 32). The understanding of this resistance mechanism is challenging due to the intracellular heterogeneity and variability of KRAS G12C-mutated cancer cells (33). Hence, to identify the crucial biomarkers involved in the sotorasib resistance, we have retrieved the RNA-seq data from the NCBI GEO database of AMG 510 treated (resistant) and untreated in KRAS G12C-mutant MIA-PaCa2 pancreatic ductal adenocarcinoma cells. The differentially expressed genes (DEGs) were identified by the linear model, and then, the DEGs were subjected to protein–protein interaction (PPI), cluster analysis, and hub gene analysis. In addition to this, the resulting probable biomarkers were also subjected to gene ontology (GO), pathway enrichment, and survival analyses to find the crucial biomarker in the sotorasib resistance.

## 2. Materials and methods

### 2.1. Data collection and pre-processing

The RNA-seq dataset retrieved for this study was accessed through NCBI Gene Expression Omnibus (GEO) database (http://www.ncbi.nlm.nih.gov/geo/). The keywords used for filtering the dataset include “KRAS mutated Pancreatic cancer” and “*Homo sapiens*” (organism). The datasets were screened, and “GSE178479” was retrieved for this study in which the sotorasib (AMG 510) resistance in the KRAS G12C-mutant MIA-PaCa2 pancreatic ductal adenocarcinoma cells was reported (34). The sequencing platform and the platform ID of the sample were “Illumina HiSeq 4000” and “GPL20301,” respectively. The number of samples used in this study was two, which includes RNA-seq profiles of AMG 510 treated (rep1 and rep2) and AMG 510 untreated (rep1 and rep2) MIA-PaCa2 cells. The present study was carried out to predict the crucial biomarkers involved in the AMG 510 resistance in pancreatic ductal adenocarcinoma cells.

The count matrix of the samples was prepared based on the matrix file information provided in the GEO database (35). The lowly expressed genes were filtered based on their counts using the counts per million (CPM) function in the *edgeR* package with the threshold of 0.5. Box plots were used to check the distribution of the read counts on the log2 scale (36). The CPM function provided the log2 counts per million which are then corrected for different library sizes. The CPM function also adds a small offset to avoid taking a log of zero. The trimmed mean of M-value (TMM) normalization was performed to eliminate composition biases between the libraries (37). This generates a set of normalization factors, where the product of these factors and the library sizes define the effective library size. The *calcNormFactors* function calculated the normalization factors between libraries.

### 2.2. Differential gene expression analysis

The *limma* package (38, 39) with the voom function was used, which transforms the read counts into logCPMs while taking account of the mean–variance relationship in the given data (40, 41). After vooming, we applied a linear model to the voom transformed data to test for differentially expressed genes (DEGs) using standard *limma* commands.

The voom transformed data have been used in *limma* to test for differential gene expression. The linear model fit was designed for each gene using the lmFit function in *limma* which estimates the groups and gene-wise variances. The contrast between the groups was then analyzed based on the makeContrasts function. Then the contrasts matrix was fitted to the object to get the statistics and estimated parameters. Here, we called the contrasts.fit function in *limma*. Furthermore, we called the eBayes function to perform the empirical Bayes shrinkage on the variances and estimated the logFC of 0.05 and their associated *p*-values. Finally, to increase the significance and reduce the false discovery rates, we used the TREAT function to predict specific genes (42–44).

### 2.3. Network analysis

The differentially expressed genes (DEGs) filtered through the TREAT function were then subjected to the STRING database (https://string-db.org/) to predict the protein–protein interactions (PPIs) with a confidence level of 0.004 and higher, and the first shell of 10 interactions was used as a filter (45). The MCODE and CytoHubba were used to analyze the probable marker genes among the DEGs (46).

### 2.4. Enrichment and survival analysis

The hub genes resulting from the network analysis were then subjected to gene ontology using the enrichGO function in the clusterProfiler package (47). The enriched biological process (BP), cellular components (CC), and molecular functions (MF) were analyzed using the enrichGO function. The KEGG pathway analysis was also carried out using the enrichKEGG function to analyze the enriched terms.

**Figure 1:**
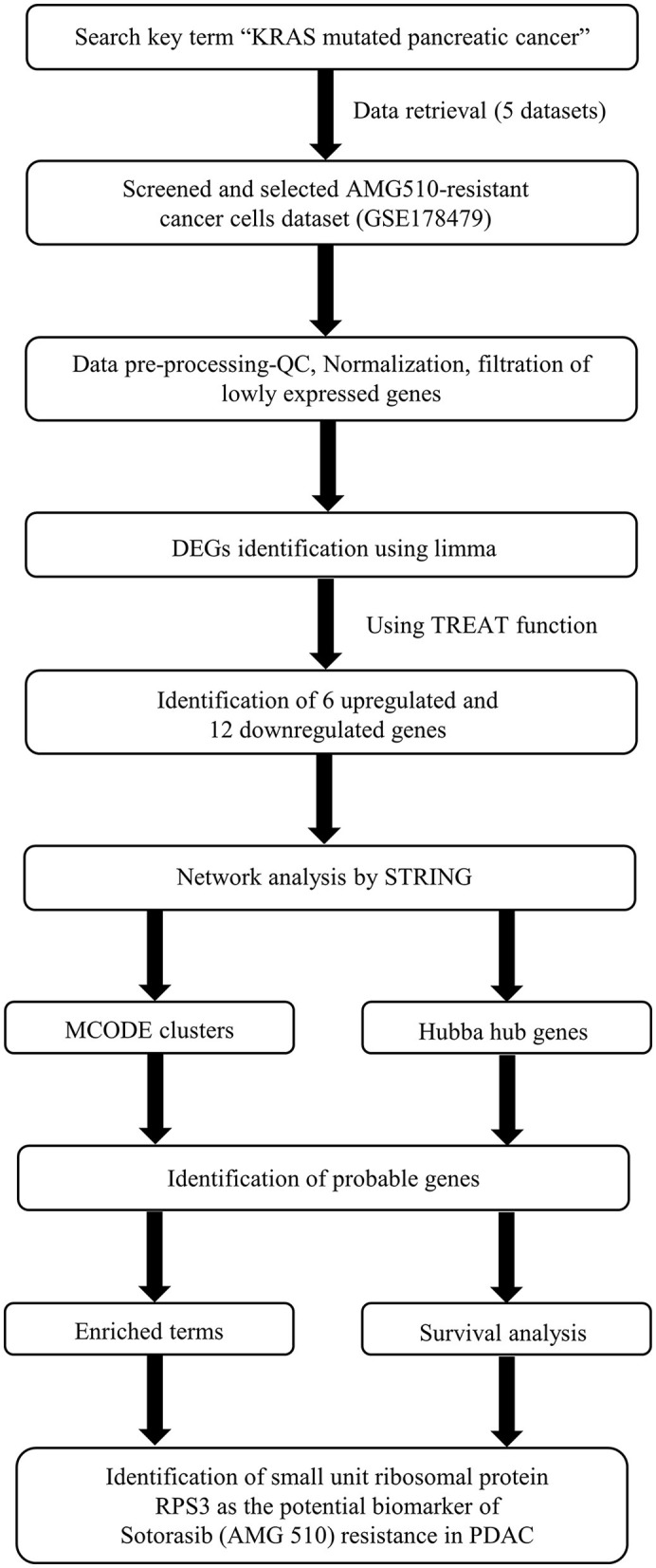
Schematic representation of the workflow of the study.

The Kaplan–Meier (KM) survival analysis was carried out based on the Spearman correlation using the Kaplan–Meier plotter online tool employing the median patient splitting mode (48, 49). Hazard is the defined slope for the survival curve which measures the incidence of death, and the hazard ratio (HR) compares the two treatment groups. If HR is 2.0, then the rate of death in one treatment group is twice the other group (50). A statistical hypothesis test was calculated based on a log-rank test. The schematic representation of the workflow of the study is shown in Figure 1.

## 3. Results

### 3.1. Identification of differently expressed genes

**Figure 2:**
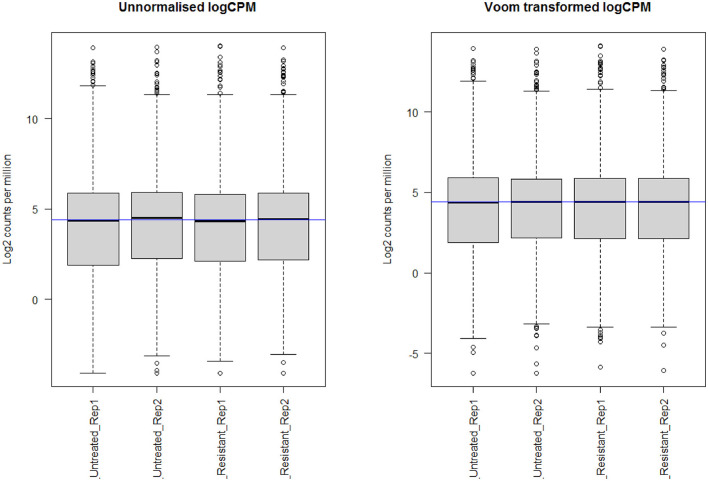
Boxplot analysis of unnormalized logCPM with the voom transformed logCPM.

Through *limma* analysis, we have tested the difference between the sotorasib (AMG 510) treated and untreated samples to analyze the genes responsible for the AMG 510 resistance in the treated group. The voom transformation of adjusting the library size with the normalization factors was analyzed through a mean–variance trend. The comparative boxplot analysis of unnormalized logCPM with the voom transformed logCPM is shown in Figure 2 which represents the precision of normalization. The CPM plot of count data after filtering the lowly expressed genes is provided in Supplementary Figure 1. The mean–variance relationship helps to analyze whether the low counts are filtered adequately and variation in the data by estimating the relationship of the log counts, which generates a precision weight for each observation and enters these into the limma empirical Bayes analysis. The voom mean–variance trend curve is shown in Supplementary Figure 2.

**Figure 3:**
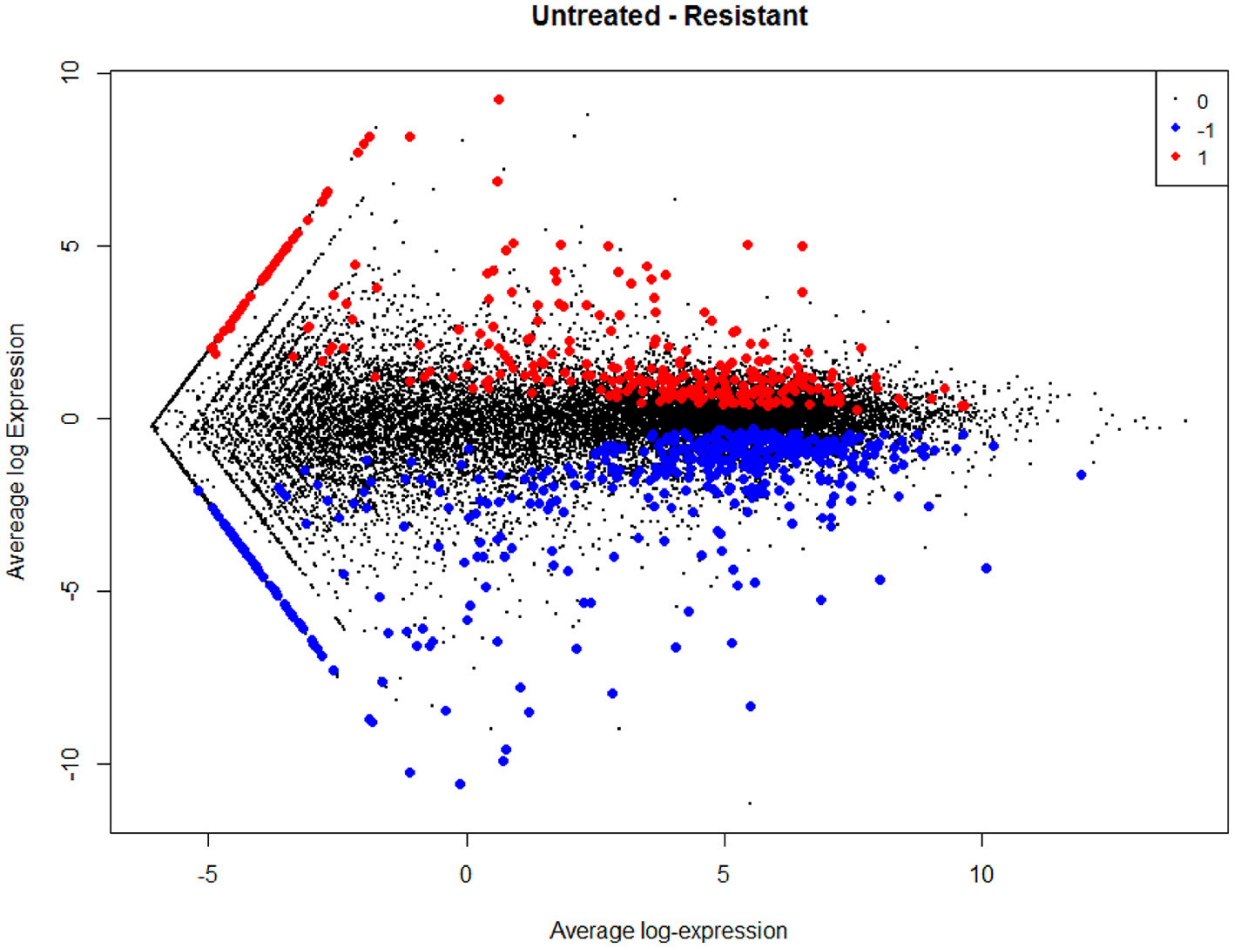
MA plot used to represent log fold change vs. mean expression between the two groups (AMG 510 treated and untreated). A scatter plot depicts the normalized mean expression on the x-axis and base-2 log fold change on the y-axis. The red dots represent the upregulated genes, the blue dots represent the downregulated genes, and the black dots represent the non-significant genes.

**Figure 4:**
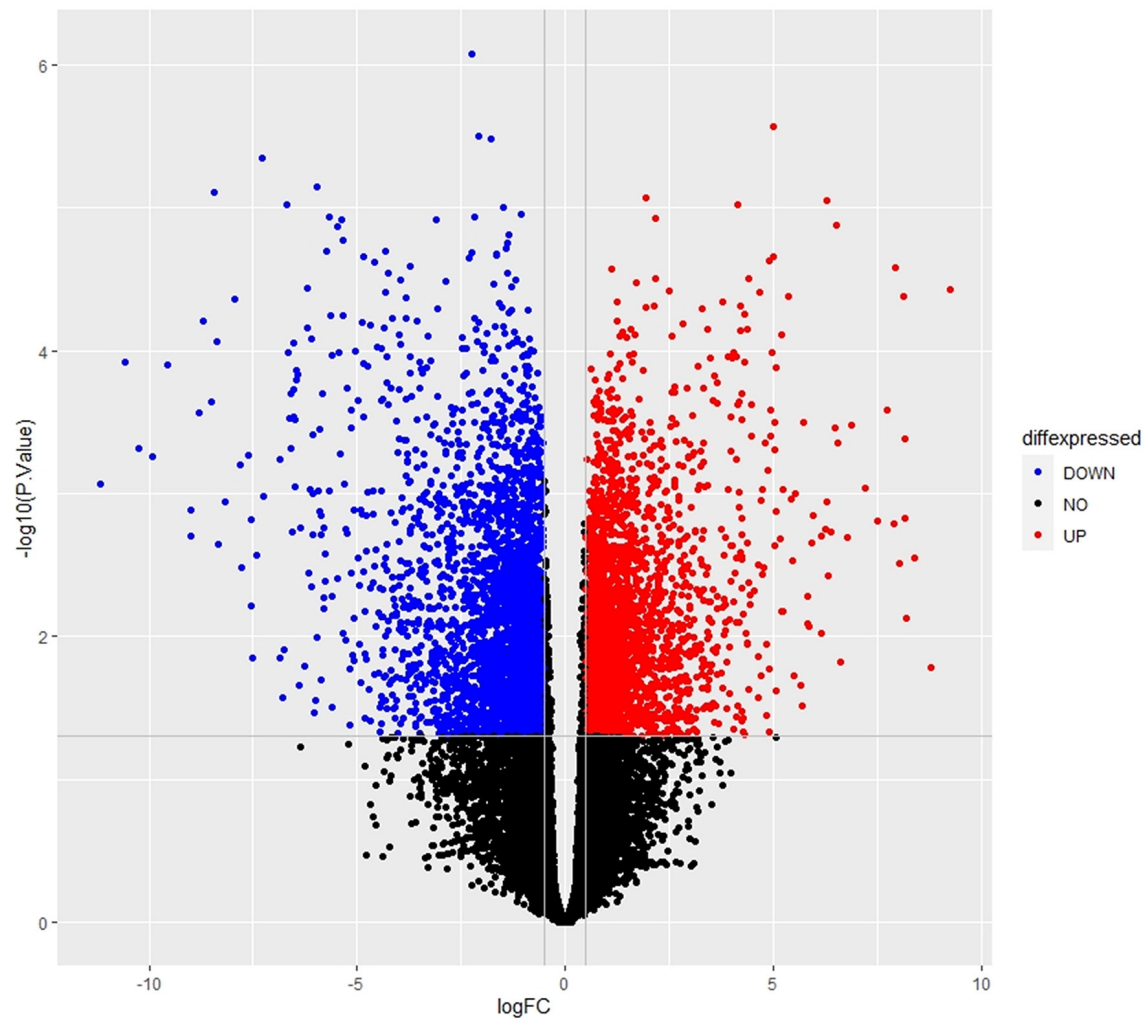
Volcano plot of the DEGs depicts the logFC on the *x*-axis and –log10 (*p*-value) on the *y*-axis. The red dots represent the upregulated genes, the blue dots represent the downregulated genes, and the black dots represent the non-significant genes.

The empirical Bayes function was used to analyze the DEGs with the linear model fit. The linear model fit resulted in the identification of upregulated and downregulated genes from the DEGs. In this study, it resulted in the differentially expressed genes among the AMG 510 treated (resistant) and untreated groups, which are repressed through the MA plot as shown in Figure 3 and the volcano plot as shown in Figure 4. Initially, the raw RNA-seq data were retrieved, pre-processed, and the differentially expressed genes (DEGs) were predicted using a cutoff on the log fold change threshold of 0.5. The *p*-value threshold of 0.05 resulted in the identification of 330 upregulated genes and 499 downregulatory genes as shown in Figure 4, and the complete list of DEGs is provided in Supplementary Table 1. To reduce false discovery rates, we further applied TREAT (*t*-tests relative to a threshold) function in the limma package, which resulted in the identification of six upregulated DEGs and 12 downregulated DEGs.

### 3.2. Network analysis

**Figure 5:**
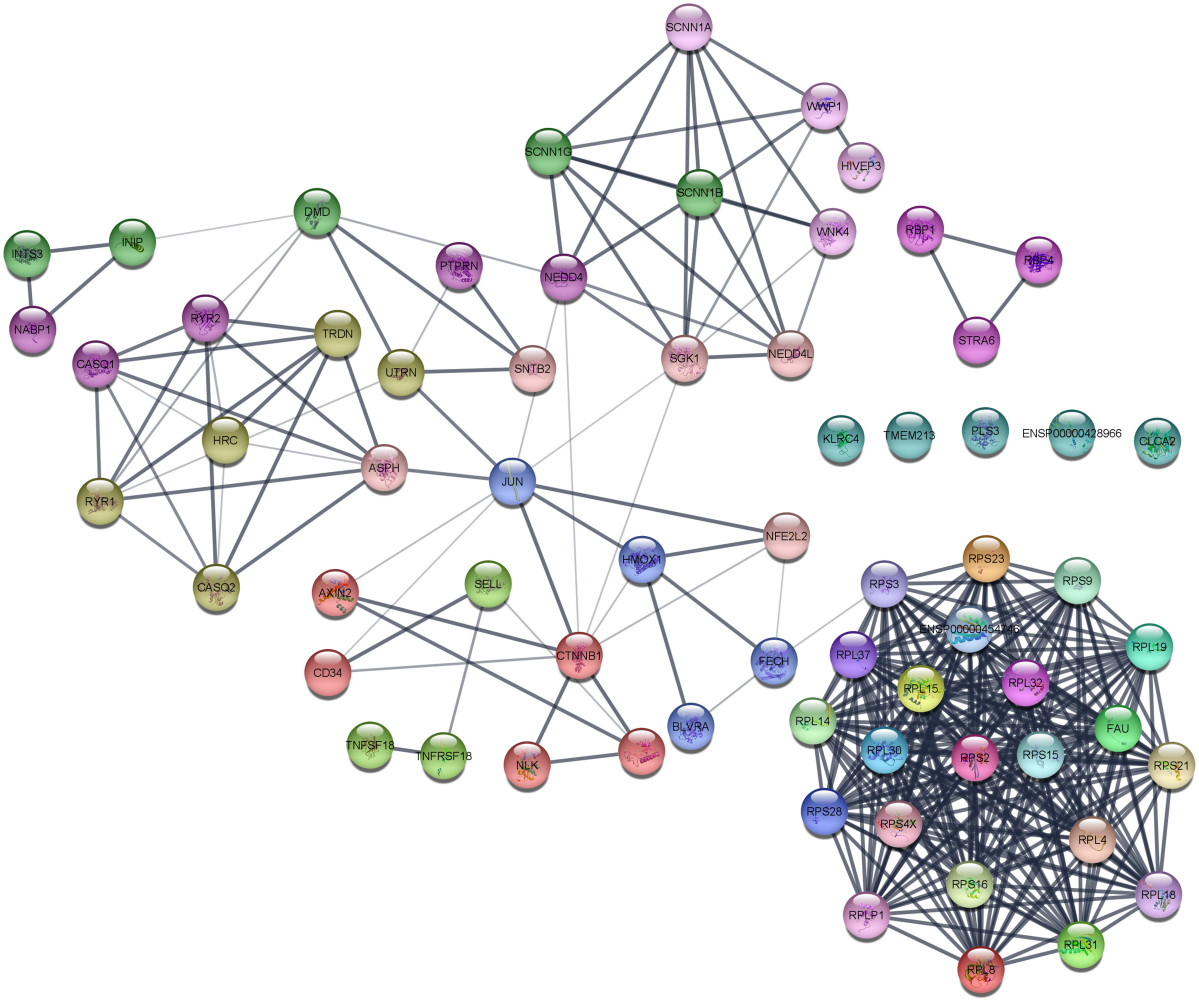
Protein–protein interaction (PPI) network of DEGs obtained from the STRING database.

The interaction network was visualized using Cytoscape using molecular complex detection (MCODE) to find the significant clusters between each node representing a gene while edges represent the interaction of the molecules. The default parameters were set including the degree cutoff of 2, node score cutoff of ≥0.2, K-score of ≥2, and max depth from seed of 100. Finally, the MCODE resulted in six clusters with the highest nodal score of 22 as shown in Figure 5.

The probable marker genes have been identified based on the highly connected nodes using CytoHubba in Cytoscape. It uses 12 scoring methods to identify the markers, namely, betweenness, bottleneck, closeness, clustering coefficient (CC), degree, the density of maximum neighborhood component (DMNC), eccentricity (EcC), edge percolated component (EPC), maximal clique centrality (MCC), maximum neighborhood component (MNC), radiality, and stress. The top 10 genes from each scoring method were isolated. Genes that are common in more than five scoring methods and also have an impact on MCODE were considered hub genes.

### 3.3. Enrichment analysis

**Figure 6:**
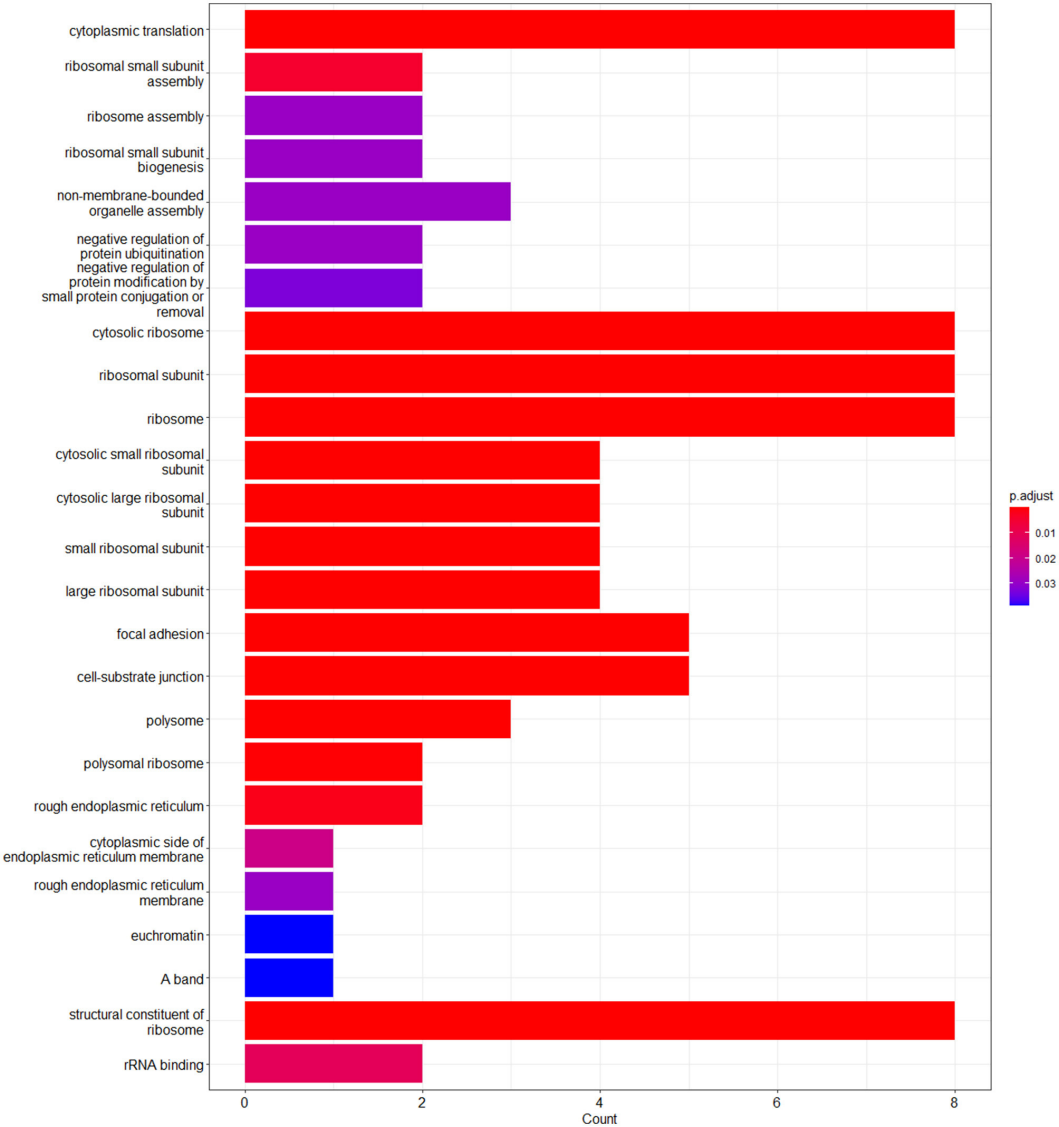
Bar plot of the enriched GO terms analyzed using enrichGO function using cluster profiler.

The enrichment analysis was performed with the GO terms: biological process (BP), cellular components (CC), and molecular functions (MF). The biological process includes cytoplasmic translation, ribosomal small subunit assembly, ribosome assembly, ribosomal small subunit biogenesis, non-membrane-bounded organelle assembly, negative regulation of protein ubiquitination, and negative regulation of protein modification by small protein conjugation or removal. Cellular components include cytosolic ribosome, ribosomal subunit, ribosome, cytosolic small ribosomal subunit, cytosolic large ribosomal subunit, small ribosomal subunit, large ribosomal subunit, focal adhesion, cell–substrate junction, polysome, polysomal ribosome, rough endoplasmic reticulum, cytoplasmic side of endoplasmic reticulum membrane, rough endoplasmic reticulum membrane, and euchromatin. Molecular functions are structural constituents of the ribosome and rRNA binding. The enriched GO terms of biological process (BP), cellular components (CC), and molecular functions (MF) are shown in Figure 6 and Table 1. Then the KEGG pathway analysis was also carried out, and the enriched term was observed as “hsa03010:Ribosome.”

**Table 1:** Gene ontology analysis of the enriched terms.

**GO term and GO ID**	**DEGs**	***p*-value**	**Adjusted *p*-value**	**Genes**
Cytoplasmic translation (GO:0002181)	BP	1.13E-16	2.91E-14	RPL4/RPLP1/RPS28/RPS9/RPL32/RPL15/RPS15/RPS3
Ribosomal small subunit assembly (GO:0000028)	BP	3.50E-05	0.004512	RPS28/RPS15
Ribosome assembly (GO:0042255)	BP	0.00037	0.029458	RPS28/RPS15
Ribosomal small subunit biogenesis (GO:0042274)	BP	0.00053	0.029458	RPS28/RPS15
Non-membrane-bounded organelle assembly (GO:0140694)	BP	0.000575	0.029458	RPS28/RPS15/RPS3
Negative regulation of protein ubiquitination (GO:0031397)	BP	0.000685	0.029458	RPS15/RPS3
Negative regulation of protein modification by small protein conjugation or removal (GO:1903321)	BP	0.000896	0.033031	RPS15/RPS3
Cytosolic ribosome (GO:0022626)	CC	3.72E-18	1.34E-16	RPL4/RPLP1/RPS28/RPS9/RPL32/RPL15/RPS15/RPS3
Ribosomal subunit (GO:0044391)	CC	3.95E-16	7.10E-15	RPL4/RPLP1/RPS28/RPS9/RPL32/RPL15/RPS15/RPS3
Ribosome (GO:0005840)	CC	4.09E-15	4.91E-14	RPL4/RPLP1/RPS28/RPS9/RPL32/RPL15/RPS15/RPS3
Cytosolic small ribosomal subunit (GO:0022627)	CC	2.30E-09	2.07E-08	RPS28/RPS9/RPS15/RPS3
Cytosolic large ribosomal subunit (GO:0022625)	CC	8.69E-09	6.26E-08	RPL4/RPLP1/RPL32/RPL15
Small ribosomal subunit (GO:0015935)	CC	1.98E-08	1.19E-07	RPS28/RPS9/RPS15/RPS3
Large ribosomal subunit (GO:0015934)	CC	1.30E-07	6.71E-07	RPL4/RPLP1/RPL32/RPL15
Focal adhesion (GO:0005925)	CC	5.12E-07	2.23E-06	RPL4/RPLP1/RPS9/RPS15/RPS3
Cell-substrate junction (GO:0030055)	CC	5.56E-07	2.23E-06	RPL4/RPLP1/RPS9/RPS15/RPS3
Polysome (GO:0005844)	CC	3.04E-06	1.10E-05	RPS28/RPL32/RPS3
Polysomal ribosome (GO:0042788)	CC	9.28E-05	0.000304	RPS28/RPL32
Rough endoplasmic reticulum (GO:0005791)	CC	0.000614	0.001842	RPL4/RPS28
Cytoplasmic side of endoplasmic reticulum membrane (GO:0098554)	CC	0.006886	0.019069	RPS28
Rough endoplasmic reticulum membrane (GO:0030867)	CC	0.011453	0.029451	RPS28
Euchromatin (GO:0000791)	CC	0.017363	0.039066	JUN
A band (GO:0031672)	CC	0.017363	0.039066	RPL15
Structural constituent of ribosome (GO:0003735)	MF	7.11E-16	3.34E-14	RPL4/RPLP1/RPS28/RPS9/RPL32/RPL15/RPS15/RPS3
rRNA binding (GO:0019843)	MF	0.000478	0.011236	RPS9/RPS3

### 3.4. Survival analysis

**Figure 7:**
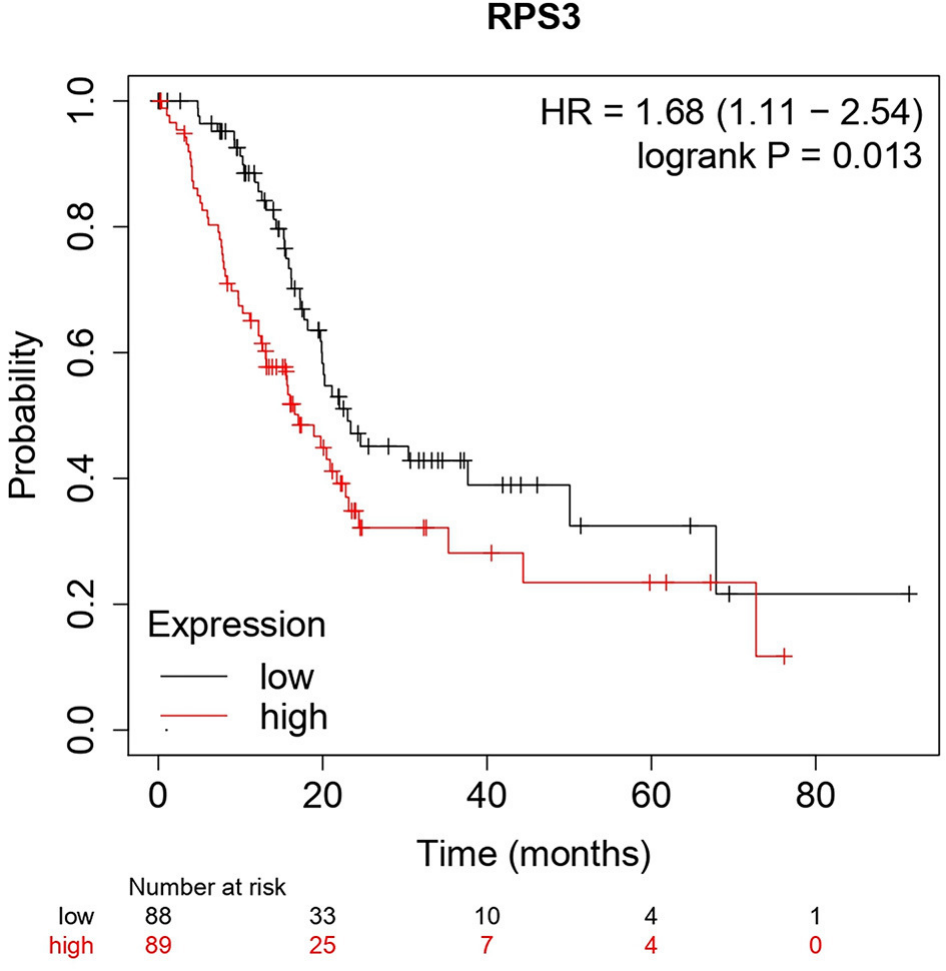
The Kaplan–Meier plot for survival analysis of key biomarkers RPS3. The *x*-axis represents the time in months, while the *y*-axis represents the probability of survival. The red and black colors represent the high expression and low expression of the biomarkers, respectively.

The Kaplan–Meier (KM) survival analysis plot was created based on Spearman's correlation, using the hazard ratio (HR) and log-rank test of the genes. In general, HR > 1 represents that the low-expression group has a higher chance of survival than the high-expression group, and HR < 1 represents that high-expression groups have a higher chance of survival than the low-expression group. The survival analysis of probable genes showed that the low expression of RPL4, RPL32, RPLP1, and RPS3 would have a higher probability for survival, and the high expression of RPS28, RPS15, RPS9, RPL15, and JUN would have a higher probability for survival. Based on the log-rank test, the significance level was set to 0.05, and if the calculated *p*-value is >0.05, the null hypothesis is retained. Based on these criteria, the ribosomal protein RPS3 was identified as a probable biomarker that showed high survival rates and *p* < 0.05 as shown in Figure 7. In addition, the HR of RPS3 is almost near two which indicates that it has twice the rate of death when compared to the others. The KM survival plots of RPL15, RPS15, RPS28, RPL4, RPL32, RPLP1, RPS9, and JUN are shown in Supplementary Figure 3.

## 4. Discussion

KRAS mutations are prevalent in many cancers including pancreatic, breast, colon, and lung with mutational hotspots at G12 (89%), G13 (9%), and Q61 (1%) residues (1, 2). The G12D, G12C, and G12V are frequent mutations with 36, 23, and 14% expressions, respectively (6). Of note, the KRAS G12C mutation is relatively high in lung adenocarcinoma than in pancreatic adenocarcinoma patients. The direct inhibition of the mutant KRAS is very prominent over other strategies but challenges the small molecule inhibitor development due to their high-affinity GTP-binding pocket and smooth surface (16, 51). Structure-based drug design guided the development and FDA approval of first-in-class potential KRAS G12C inhibitor sotorasib (AMG 510) that has changed the scenario in which the mutant KRAS became undruggable (26). Recently, in December 2022, FDA granted the accelerated approval for Adagrasib (MRTX849) for the treatment of KRAS G12C-mutated NSCLC (30). In addition to this, several pharma industries have initiated to design and develop novel KRAS mutant inhibitors (mutant specific/pan-KRAS). Several KRAS G12C (GDP-bound off state) inhibitors, such as sotorasib (AMG 510), adagrasib (MRTX849), GDC-6036, JNJ-74699157, D-1553, JDQ443, LY3537982, LY3499446, ARS1620, and KRAS G12C (GDP-bound off state) inhibitors such as RMC-6291, RMC-6236, and RM-018, and Pan KRAS Switch I/II inhibitors such as BI-2852, are being studied in preclinical and clinical studies (18, 52–55). Recent pieces of evidence report the resistance to AMG 510 among KRAS G12C-mutant cancer patients (31, 33). Moreover, Adagrasib (MRTX849) and ARS1620 were reported to have acquired resistance in KRAS G12C-mutant cells (33, 56). Amplification of the mesenchymal epithelial transition factor receptor (MET); activating mutations of downstream effectors, such as BRAF, and dual specificity mitogen-activated protein kinase kinase 1 (MEK1); oncogenic fusion with fibroblast growth factor receptor 3 (FGFR3) and CCDC6-RET; and loss-of-function mutations of phosphatase and tensin homolog (PTEN) and neurofibromin 1 (NF1) were reported to be the key elements involved in the resistance mechanisms to KRAS mutant inhibitors in lung adenocarcinoma and colorectal adenocarcinoma (56, 57). Unlike the abovementioned resistance mechanisms, our results revealed a significant correlation between the sotorasib resistance in KRAS G12C-mutant cells and ribosomopathies.

Recently Chan et al. (34) reported an interesting study on the identification of sotorasib (AMG 510) resistance in the KRAS G12C-mutant MIA-PaCa2 pancreatic ductal adenocarcinoma cells when treated with increasing dosage (0.1–5 μM) for 60 days and found that MIA-PaCa2 showed resistance at 5 μM treatment of AMG 510 (34). This interested us to identify the crucial biomarkers involved in the AMG 510 resistance in the KRAS G12C-mutant MIA-PaCa2 pancreatic ductal adenocarcinoma cells. In addition to MIA-PaCa2 cells, they have also tested the AMG 510 resistance in SW1463 human Caucasian rectum adenocarcinoma, LU99 lung giant cell carcinoma, and LU65 lung carcinoma cell lines which have KRAS G12C mutations.

The main aim of the present study was to identify the key biomarker genes involved in the AMG 510 resistance. Initially, the raw RNA-seq data were retrieved, pre-processed, and the differentially expressed genes (DEGs) were predicted which resulted in the identification of 330 upregulated genes and 499 downregulatory genes as shown in Figure 4 and Supplementary Table 1. The *t*-tests relative to a threshold (TREAT) function reduced the false discovery rates of DEGs (42), which further resulted in the identification of six upregulated and 12 downregulated genes. These filtered DEGs were studied for the protein–protein interaction network using STRING which resulted in four MCODE clusters, and the MCODE cluster 1 showed the highest nodal density among the other clusters as shown in Figure 5. In addition, cluster analysis and hub gene analysis were carried out which resulted in probable biomarkers as shown in Figure 6, and the enriched GO terms of biological process (BP), cellular components (CC), and molecular functions (MF) are shown in Table 1. In general, HR > 1 represents that the low-expression group has a high chance of survival than the high-expression group, and HR < 1 represents that the high-expression group has a high chance of survival than the low-expression group (58). Finally, the survival analysis based on the hazard ratio and log-rank test resulted in the identification of RPS3 as the probable biomarker with high survival rates and *p* < 0.05 as shown in Figure 7. Based on the log-rank test, the significance level was set to 0.05, and if the calculated *p*-value is >0.05, the null hypothesis is retained. Moreover, the HR of RPS3 is nearly 2 which indicates that it has twice the rate of death when compared to the others. The KM survival plots of RPL15, RPS15, RPS28, RPL4, RPL32, RPLP1, RPS9, and JUN are shown in Supplementary Figure 3. In addition, the GO of all the 330 upregulated genes and 499 downregulatory genes shown in Supplementary Table 1 reveals that the myc transcriptional targets, such as E2F transcription factor 6 (ENSG00000169016), are upregulated and the CDK10 (ENSG00000185324) is downregulated. Generally, the E2F6 regulates the gene expression of proteins involved in cell proliferation and the CDK10 acts as a tumor suppressor. Furthermore, the CDC25B (ENSG00000101224) expression has a p53-dependent tumor suppressive effect, which is downregulated. The anti-apoptotic BCL-6 (ENSG00000113916) is downregulated. The abovementioned targets are also involved in the RAS signaling pathway. These data suggest that the resistance could be a result of RNA pol I machinery hyperactivation and apoptosis evasion. The present study revealed that the small unit ribosomal protein RPS3 is known to be only expressed in the AMG 510 resistant MIA-PaCa2 cells and identified as a significant biomarker involved in the resistance of AMG 510. These novel identifications resulted from the emergence and accumulation of RNA-Seq data of drug-resistant cancer cells.

Ribosome biogenesis starts from the nucleolus and ends in the cytoplasm with the formation of the mature ribosome from rRNA and ribosomal proteins (59). In normal cells, the RNA pol I initiates the Pol I transcription followed by the pre-rRNA processing and modification and then assembled with ribosomal proteins (RPs) to form mature 60s and 40s subunits and ultimately takes part in protein synthesis. Unlike normal cells, the RNA pol I is hyperactivated leading to the altered rRNA modifications and altered RPs extraribosomal functions, thus forming the onco-ribosomes and translating the oncogenic mRNAs and ultimately ending with ribosomopathies (59). Some large subunit ribosomal proteins, such as RPL5, RPL9, RPL10, RPL11, RPL15, RPL21, RPL22, RPL23A, RPL27, RPL31 RPL34, RPL35, RPL36, and large subunit ribosomal proteins, such as RPS7, RPS15, RPS15A, RPS17, RPS19, RPS20, RPS24, RPS27, and RPSA, are reported to have significant roles in the progression of various types of cancers including lung, colon, breast, and pancreatic cancers (60–62). Generally, the ribosomal proteins (RPs) directly/indirectly interact with the Mdm2/Mdm4 E3 ubiquitin-protein ligases, which in turn regulate the degradation of p53 tumor suppressor protein resulting in the tumor progression (62, 63). An interesting study reports that the WD repeat-containing protein 74 (WDR74) alters the RPL5 levels and promotes metastasis by degrading p53 via the RPS15-Mdm2 axis in lung carcinoma (64). The ribosomal proteins were upregulated in KRAS mutant Panc-1 cells, and their inhibition results in cell cycle arrest, apoptosis induction, and antiproliferation (65, 66).

RPS3 knockdown in Caco-2 colon cancer cells showed decreased cancer progression and increased apoptosis via p53 upregulation and reduced activity of lactate dehydrogenase (LDH) (67). RPS3 was also reported to induce apoptosis by disrupting its interaction with E2F1 and also upregulates the expression of pro-survival genes in NSCLC (68). On this note, the mutations in the ribosomal proteins are also highly involved in tumorigenesis. The RPs were reported to interact with MDM2/4 and inhibit p53, and overexpression was observed as a result of the hyperactivation of RNA polymerase I machinery. The inhibition of RNA polymerase I machinery by inhibitors, such as CX-3543 and CX-5461, promotes p-53-dependent apoptosis in several cancers (69, 70). The clinical trials of RNA polymerase I machinery by inhibitors CX-5461 (NCT02719977) and CX-3543 (NCT00955786) resulted in the identification of safety, tolerable dosage, and effective dosage regimes and also resulted in less toxicity in patients (71). The potential of individual RNA polymerase I machinery inhibitors was studied, and combination strategies have to be studied in near future from the successful interventions from preclinical studies. Chan et al. (34) reported that the sotorasib resistance was offered by the PAK/PI3K pathway in KRAS G12C-mutant MIA-PaCa2 cells, and our bioinformatics analysis showed that RPS3 was the crucial biomarker. Recent reports show that RPS3 mediates the PI3K-Akt signaling axis in cancer cells, which correlates with our findings from the study (72, 73).

From the above understandings, we observe and conclude that the small unit ribosomal protein RPS3 is the crucial biomarker of the AMG 510 resistance in KRAS G12C-mutant MIA-PaCa2 cell pancreatic ductal adenocarcinoma cells. The study outcomes and the possible future directions to combat the Sotorasib resistance in KRAS G12C mutant cells were shown in the Graphical Abstract. Co-targeting of ribosomal proteins along with the target-specific inhibitors (here KRAS G12C-mutant inhibitor) will pave way for the development of precision treatment, such as using CRISPR-Cas and T-cell immunotherapy, in cancer.

## 5. Conclusion

The current study was performed to evaluate the crucial biomarkers involved in the KRAS G12C inhibitor, sotorasib (AMG 510). From the analysis, we finally conclude that the ribosomal protein RPS3 is the crucial biomarker involved in the AMG 510 resistance in the KRAS G12C-mutant MIA-PaCa2 cell pancreatic ductal adenocarcinoma cells. From the study results and previous literature, we also report that resistance could result from the degradation of p53 via the RPs-MDM2/MDM4-p53 axis. Thus, the combinatorial treatment strategy of (i) KRAS G12C-mutant inhibitors and (ii) RNA polymerase I machinery inhibitors, such as CX-3543 and CX-5461, could be a possible strategy to tackle resistance and has to be studied in *in vitro* and *in vivo* settings, which promotes the increased therapeutic treatment of KRAS G12C-mutated cancers in the era of precision medicine.

## Data availability statement

Publicly available datasets were analyzed in this study. This data can be found here: GSE178479.

## Author contributions

PSR conceptualized and designed the study. PSR and AP retrieved the data, carried out all the analyses, and wrote the manuscript. All the results were validated and the manuscript was corrected by IE and SA. All authors proofread the manuscript. All authors contributed to the article and approved the submitted version.

## Supplementary material

Supplementary Figure 1 CPM plot of count data after filtering the poorly expressed genes.

Supplementary Figure 2 Voom mean–variance trend curve. It depicts that the lowly expressed genes are filtered properly. t. Counts nearly 0 (plot *x*-axis value −1) have low standard deviations. This rises immediately for low counts and then gradually decreases.

Supplementary Figure 3 Kaplan–Meier plot for survival analysis of RPL4 **(A)**, RPL32 **(B)**, RPLP1 **(C)**, RPS9 **(D)**, JUN **(E)**, RPL15 **(F)**, RPS15 **(G)**, and RPS28 **(H)**. The *x*-axis represents the time in months, while the *y*-axis represents the probability of survival. The red and black colors represent the high expression and low expression of the biomarkers, respectively.
