# Supplementary figures and images for: Potential biomarkers uncovered by bioinformatics analysis in sotorasib resistant-pancreatic ductal adenocarcinoma
Source: Front Med (Lausanne). 2023 Jun 15;10:1107128. doi: 10.3389/fmed.2023.1107128 (PMC10310804; doi:10.3389/fmed.2023.1107128)

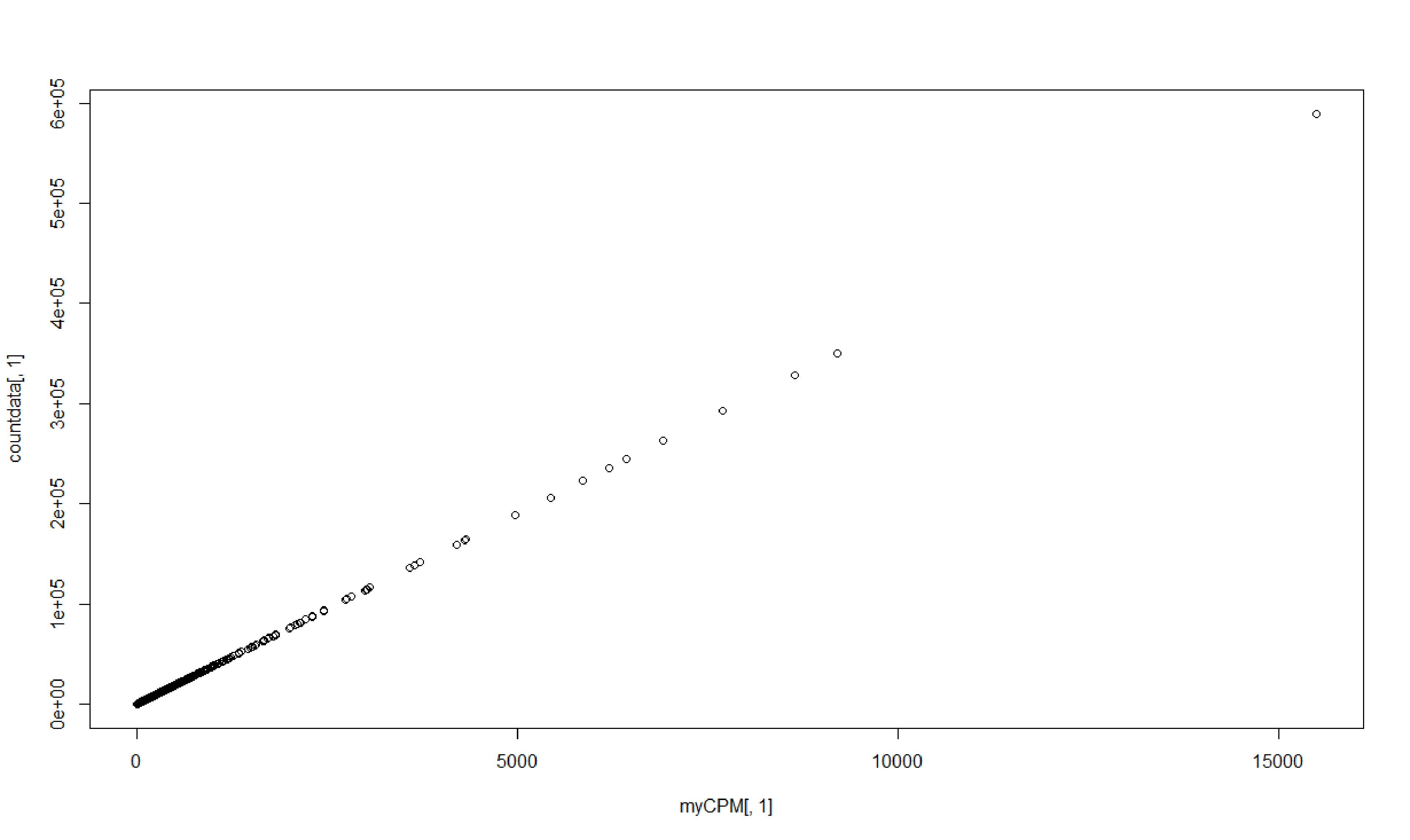

Supplement: Supplementary Figure 1 — CPM plot of count data after filtering the poorly expressed genes. [file Image_1.TIF]

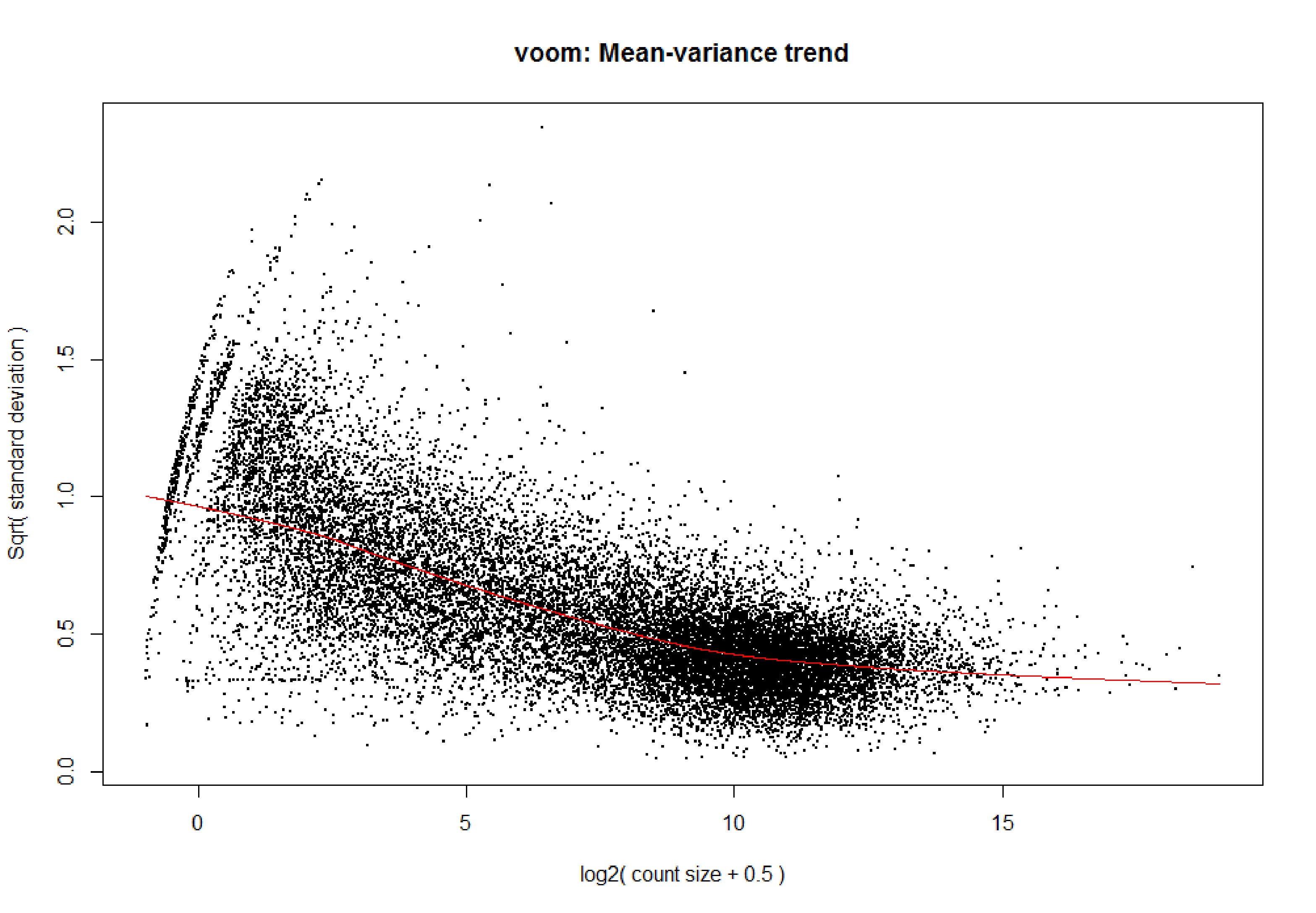

Supplement: Supplementary Figure 2 — Voom mean–variance trend curve. It depicts that the lowly expressed genes are filtered properly. t. Counts nearly 0 (plot x-axis value −1) have low standard deviations. This rises immediately for low counts and then gradually decreases. [file Image_2.TIF]

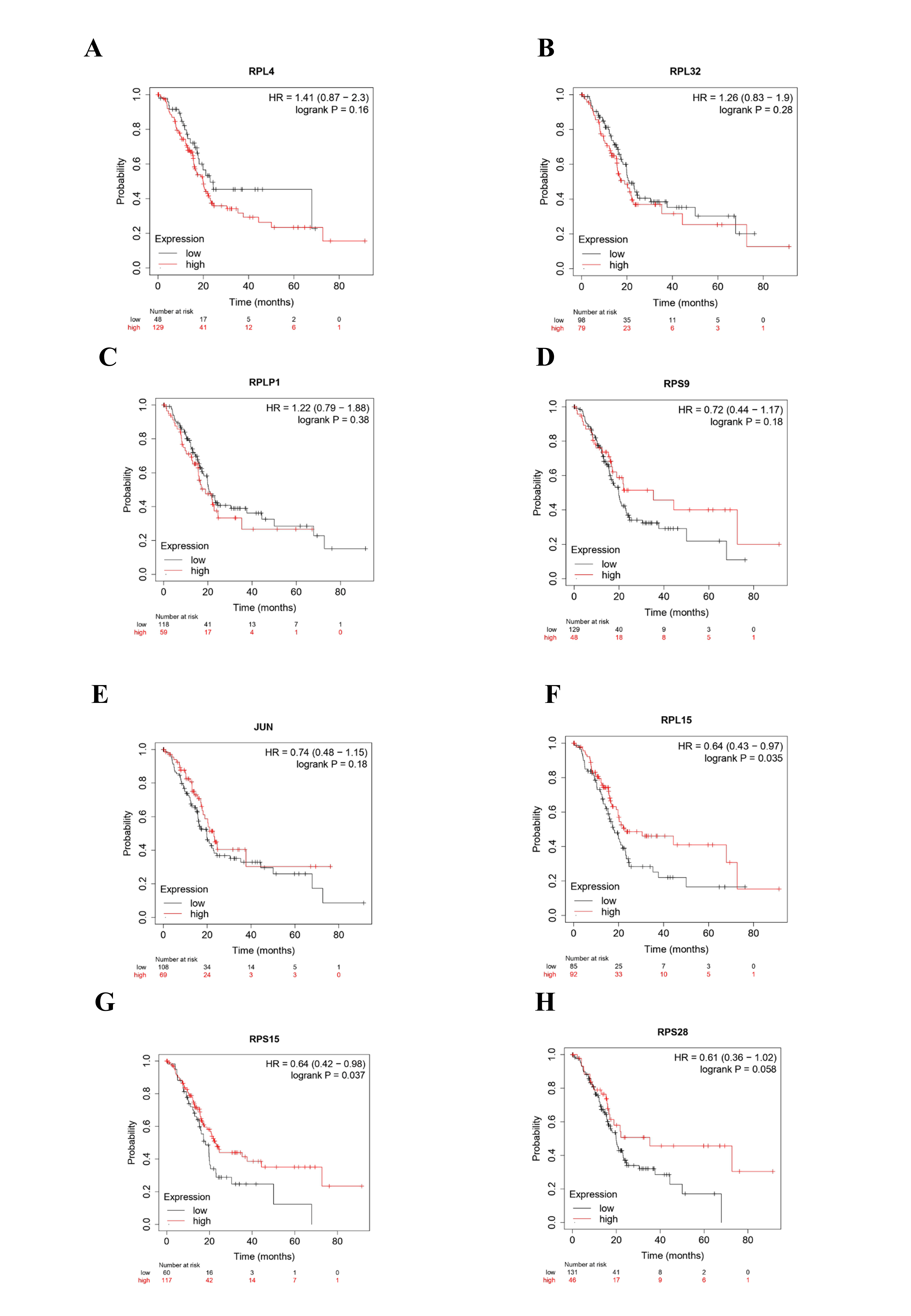

Supplement: Supplementary Figure 3 — Kaplan–Meier plot for survival analysis of RPL4 (A), RPL32 (B), RPLP1 (C), RPS9 (D), JUN (E), RPL15 (F), RPS15 (G), and RPS28 (H). The x-axis represents the time in months, while the y-axis represents the probability of survival. The red and black colors represent the high expression and low expression of the biomarkers, respectively. [file Image_3.TIF]
